# Expression Pattern of PDE4B, PDE4D, and SFRP5 Markers in Colorectal Cancer

**DOI:** 10.3390/medicina60081202

**Published:** 2024-07-25

**Authors:** Mateo Bevanda, Nela Kelam, Anita Racetin, Natalija Filipović, Daniela Bevanda Glibo, Ivana Bevanda, Katarina Vukojević

**Affiliations:** 1 Department of Surgery, School of Medicine, University of Mostar, University Hospital Mostar, Bijeli Brijeg bb, 88000 Mostar, Bosnia and Herzegovina; bevanda333@gmail.com; 2 Laboratory for Early Human Development, Department of Anatomy, Histology and Embryology, University of Split School of Medicine, Šoltanska 2A, 21000 Split, Croatia; nela.kelam@mefst.hr (N.K.); anita.muic@mefst.hr (A.R.); natalija.filipovic@mefst.hr (N.F.); 3 Center for Translational Research in Biomedicine, University of Split School of Medicine, Šoltanska 2A, 21000 Split, Croatia; 4 Department of Gastroenterology, School of Medicine, University of Mostar, University Hospital Mostar, Bijeli Brijeg bb, 88000 Mostar, Bosnia and Herzegovina; ela.bevanda@gmail.com; 5 Department of Endocrinology, School of Medicine, University of Mostar, University Hospital Mostar, Bijeli Brijeg bb, 88000 Mostar, Bosnia and Herzegovina; bjelanovic.ivanaaa@yahoo.com

**Keywords:** colorectal cancer, PDE4B, PDE4D, SFRP5

## Abstract

*Background and Objectives:* Colorectal cancer (CRC) is the most frequently diagnosed malignant disease of the gastrointestinal system, and new diagnostic and prognostic markers are needed to elucidate the complete tumor profile. *Materials and Methods*: We used CRC tumor tissues (Dukes’ A-D) and adjacent noncancerous tissues of 43 patients. Immunohistochemistry was used to examine the expression of phosphodiesterase 4B (PDE4B), phosphodiesterase 4D (PDE4D), and secreted frizzled related protein 5 (SFRP5) markers. We also analyzed the expression levels of *PDE4B*, *PDE4D*, and *SFRP5* in CRC tissues compared to control tissues using RNA-sequencing data from the UCSC Xena browser. *Results*: In CRC stages, the distribution of PDE4B-positive cells varied, with differing percentages between epithelium and lamina propria. Statistically significant differences were found in the number of PDE4B-positive epithelial cells between healthy controls and all CRC stages, as well as between different CRC stages. Similarly, significant differences were observed in the number of PDE4B-positive cells in the lamina propria between healthy controls and all CRC stages, as well as between different CRC stages. CRC stage Dukes’ C exhibited a significantly higher number of PDE4B-positive cells in the lamina propria compared to CRC stage Dukes’ B. Significant differences were noted in the number of PDE4D-positive epithelial cells between healthy controls and CRC stages Dukes’ A, B, and D, as well as between CRC stage Dukes’ C and stages A, B, and D. CRC stage Dukes’ A had significantly more PDE4D-positive cells in the lamina propria compared to stage D. Significant differences were also observed in the number of SFRP5-positive cells in the lamina propria between healthy controls and all CRC stages, as well as between CRC stages Dukes’ A and D. While the expression of PDE4D varied across CRC stages, the expression of SFRP5 remained consistently strong in both epithelium and lamina propria, with significant differences noted mainly in the lamina propria. The expression levels of *PDE4B*, *PDE4D*, and *SFRP5* reveal significant differences in the expression of these genes between CRC patients and healthy controls, with notable implications for patient prognosis. Namely, our results demonstrate that *PDE4B, PDE4D,* and *SFRP5* are significantly under-expressed in CRC tissues compared to control tissues. The Kaplan–Meier survival analysis and the log-rank (Mantel–Cox) test revealed distinct prognostic implications where patients with lower expression levels of *SFRP5* exhibited significantly longer overall survival. The data align with our immunohistochemical results and might suggest a potential tumor-suppressive role for these genes in CRC. *Conclusions*: Considering significantly lower gene expression, aligned with our immunohistochemical data in tumor tissue in comparison to the control tissue, as well as the significantly poorer survival rate in the case of its higher expression, we can hypothesize that SFRP5 is the most promising biomarker for CRC out of the observed proteins. These findings suggest alterations in PDE4B, PDE4D, and SFRP5 expression during CRC progression, as well as between different stages of CRC, with potential implications for understanding the molecular mechanisms involved in CRC development and progression.

## 1. Introduction

Colorectal cancer (CRC) is considered the most frequently diagnosed malignant disease of the gastrointestinal system, with a frequency of 10.2% and a mortality rate of 9.2% of all cancers [1,2]. It is not a homogeneous disease but can be classified into different subtypes characterized by specific molecular and morphological changes. Around 95% of all cases of CRC are adenocarcinomas, and the rest are neuroendocrine tumors and small-cell carcinoma [3]. A major feature of CRC is genetic instability, which can arise through at least two distinct mechanisms. The most common (about 84%) is characterized by chromosomal instability, with major changes in the number and structure of chromosomes, including deletions, translocations, and other chromosomal changes [4]. The second group (around ~13–16% of sporadic CRC) is characterized by hypermutation and microsatellite instability (MSI) due to defective DNA mismatch repair (MMR) [5]. Despite advances in diagnosis and new therapeutic options, the clinical outcomes of patients with colorectal cancer are still unsatisfactory. Overall, patient survival largely depends on the stage of the disease at the time of diagnosis and/or surgical resection [6,7,8]. The most important prognostic indicator is the expansion of the tumor at the time of diagnosis. It is necessary to determine the exact stage of the tumor according to the TNM system or the established system introduced by Dukes. TNM is a system of classification primarily used for solid tumors, and it is based on assessing the tumor, regional lymph nodes, and distant metastasis [9]. Mark T—Tumor describes the size of the primary tumor and its invasion into adjacent tissues; N—Nodes describe the tumor’s regional lymph node involvement; M—Metastasis is used to identify the presence of distant metastases of the primary tumor [9]. The Dukes classification includes four grades based on the extent of tumor spread. Stage A represents tumors confined to the rectal wall. The tumor is in the inner lining of the bowel or slightly growing into the muscle layer. Stage B represents tumors that have penetrated the wall of the rectum, where the muscle layer is occupied. Stage C represents tumors with metastases in at least one lymph node close to the bowel. Stage D represents tumors with metastases in lymph nodes and distant organs [6,10,11]. The five-year survival for stage Dukes’ A is more than 90% and only 5% for Dukes’ stage D [3]. However, molecular classifications and epithelial–mesenchymal transition (EMT) concepts have become more important today, and novel markers are needed to elucidate the complete molecular tumor profile [12].

Phosphodiesterases (PDEs) are categorized as metalloproteinases specialized in breaking down the secondary messengers’ cyclic adenosine monophosphate (cAMP) and guanosine 3′,5′-cyclic monophosphate (cGMP) [13,14]. The phosphodiesterase-4 (PDE4) family of enzymes is the most prevalent PDE in the immune cells, epithelial cells, and brain cells and manifests as an intracellular non-receptor enzyme that modulates inflammation and epithelial integrity [15,16]. Blocking PDE4 is predicted to induce various outcomes by increasing cAMP levels, leading to the control of numerous genes and proteins [15]. Phosphodiesterase 4D (PDE4D) degrades cAMP and has recently been identified as an oncogene in different human cancer types [17]. Additionally, PDE4D has emerged as a novel tumor-promoting molecule, presenting a distinctive targetable enzyme across multiple human cancers, including lung, prostate, melanoma, ovarian, endometrial, and gastric cancers [18]. An important paralog of this gene is phosphodiesterase 4B (PDE4B). PDE4B plays a role in behaviors associated with dopamine and stress [6]. Decreasing the activity of PDE4B in mouse models enhances memory and long-term plasticity, suggesting potential for therapeutic uses [19]. Additionally, PDE4B inhibition may be a viable treatment to reduce inflammation [19].

Secreted frizzled related proteins (SFRPs) are antagonists of the wingless-related integration site (Wnt) signaling pathway that binds directly to Wnt ligands and thus prevent their binding to frizzled receptors [20]. There are currently eight known family members of SFRPs [20]. Expression of SFRP has been observed in many malignant cancers [21]. SFRP5 is known to be associated with hepatocellular carcinoma and gastric cancer [14,22]. Nonetheless, the expression and importance of PDE4D, PDE4B, and SFRP5 in CRC remain unclear. Continued prevention, early detection, and treatment efforts remain essential in combating CRC and reducing its negative impact on public health.

## 2. Materials and Methods

### 2.1. Ethics

This study was approved by the Ethics Committee of the University of Mostar School of Medicine in accordance with the Helsinki Declaration (approval number 1271723 from 2 February 2023).

### 2.2. Tissue Procurement and Immunohistochemistry

Forty-three tumor tissue samples (12 of Dukes’ A, 11 of Dukes’ B, 10 of Dukes’ C, and 10 of Dukes’ D; Table 1) were obtained from the Department of Pathology, Cytology, and Forensic Medicine at the University Hospital Mostar. Inclusion criteria encompassed patients with CRC older than 18 years, of both sexes and all cancer stages. Stratified randomization was used to ensure a balanced distribution of key characteristics (cancer stage) across treatment groups. Recruitment was conducted in collaboration with healthcare practitioners to identify eligible patients, supplemented by outreach through clinical settings. Participants were approached through direct contact during medical appointments, supplemented by follow-up communication and informational sessions to ensure thorough understanding and informed consent. The participants in this study did not receive any medications prior to the commencement of the study, since this was their first CRC diagnosis.

Inclusion criteria for samples collected necessitated having an adequate amount of paraffin block material for immunohistochemistry (IHC) and complete clinical data. Exclusion criteria involved incomplete laboratory results, insufficient material for IHC, and lack of control tissue. Macroscopic examination and measurement of the tumor samples were conducted. Each sample contains tumor tissue and healthy control tissue. The tissues were dehydrated in increasing concentrations of ethanol solutions and embedded in paraffin. Paraffin blocks were cut using a microtome (Leica RM2155, Pittsburgh, PA, USA) in 4 μm sections to improve staining quality, reduce cell overlap, provide fewer artifacts, and enhance the clarity of microphotographs for data analysis. The immunohistochemistry protocol was followed as described previously [23]. Briefly, the tissue sections were deparaffinized in xylene, rehydrated in ethanol solutions of decreasing concentrations and briefly rinsed in distilled water. The sections were heated in a citrate buffer (pH 6) for 15 min at 100 °C in a steam bath (Tefal, Minicompact, Rumilly, Haute-Savoie, France) and cooled to room temperature. Afterward, the tissue sections were incubated with a blocking buffer (ab64226, Abcam, Cambridge, UK) for 20 min and then with the appropriate combination of primary antibodies for one hour (Table 2).

### 2.3. Data Acquisition and Analysis

The hematoxylin and eosin slides were examined using a light microscope (BX40, Olympus, Tokyo, Japan). Samples were observed using a BX51 microscope (Olympus, Tokyo, Japan) at either 20× or 40× objective magnification and captured with a DP71 digital camera (Olympus, Tokyo, Japan). PDE4B, PDE4D, and SFRP5-positive cell counts were conducted in the regions of interest for all studied groups and delineated based on their distinct morphological characteristics, including epithelium and lamina propria. Cells were categorized as either positive (stained) or negative (unstained). Microphotographs were processed and analyzed using the ImageJ program (National Institutes of Health, Bethesda, MD, USA). The captured images were divided into squares measuring 20 μm × 20 μm at ×40 magnification to facilitate precise data collection and analysis. Positive and negative cells were counted as described previously [24]. Briefly, only squares completely encompassing the region of interest were checked. To prevent duplicate counting of cells, every alternate section was utilized, and cells below the left and upper boundaries of each square were disregarded, with only those along the right and lower borders considered. The percentage of positive cells was computed across ten representative fields from each image, presented as mean ± standard deviation (SD), and compared across the regions of interest. Adobe Photoshop (Adobe Photoshop CS., 2004, Berkeley, CA, USA: Peachpit Press) was used for image assembly. Captured images in their original size at 40× magnification were used for the presentation of the images to better illustrate and showcase the overall morphology and spatial organization of the positive cells within the epithelium and lamina propria of the tissue examined.

### 2.4. Semi-Quantification

The intensity of staining of the normal colonic mucosa and CRC tumor tissue was semi-quantitatively evaluated into four groups according to the staining reactivity: no reactivity = −, mild reactivity = +, moderate reactivity = ++, and strong reactivity = +++.

### 2.5. Statistical Data Analysis

The GraphPad software was used for statistical data processing. To compare the expression of observed proteins in different substructures of colon mucosa (lamina propria and epithelium) between the control tissue and CRC stages, ordinary one-way ANOVA followed by Tukey’s multiple comparison tests was used. Within each observed group (control tissue and each examined CRC stage), a two-way ANOVA test followed by Šídák’s multiple comparisons test was used to compare the expression of the observed proteins between the different substructures (lamina propria and epithelium). Data were presented as mean ± standard deviation. A statistically significant difference was set at *p* < 0.05.

### 2.6. Transcriptomics Data Analysis

We used TCGA Colon and Rectal Adenocarcinoma (COADREAD) gene expression by the RNAseq (polyA + IlluminaHiSeq) dataset, downloaded from the Xena database (University of California Santa Cruz) (https://xenabrowser.net/datapages/; accessed on 4 May 2023). The dataset is combined from TCGA colon adenocarcinoma and rectum adenocarcinoma, with no history of neoadjuvant treatment, measured using the Illumina HiSeq 2000 RNA Sequencing platform at the University of North Carolina TCGA genome characterization center. This dataset shows the gene-level transcription estimates, as in log2(x + 1)-transformed RSEM normalized counts. Overall survival and gene expression of samples were exported as text and edited in Microsoft^®^ Excel^®^ 2019 MSO version 2305 (Microsoft Corp., Redmond, WA, USA). After data curation for double samples and samples that did not contain data for expression of studied factors, 431 patient samples remained for the analysis, of which 380 were primary tumors (PT), and 51 were normal solid tissue from the healthy resection margins (STN). Using GraphPad 9.0.0. software (GraphPad Software, San Diego, CA, USA), an unpaired *t*-test was used to compare the expression of *PDE4B*, *PDE4D,* and *SFRP5* genes between tumor and solid tissue normal specimens.

Survival analysis based on expression quartiles for each gene was completed using GraphPad software. The Kaplan–Meier method and the log-rank test were used for statistical analysis of the survival length. A statistically significant difference was set at *p* < 0.05.

## 3. Results

### 3.1. General Characteristics of CRC Visualized by Hematoxylin and Eosin Staining

**Figure 1:**
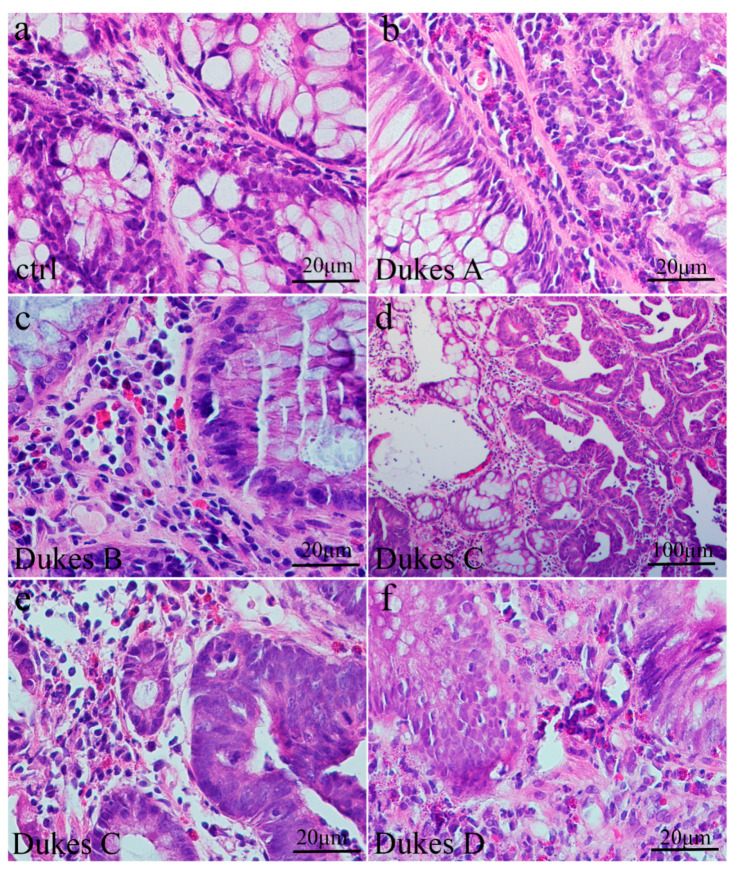
Section through the normal adult colon (**a**) showing crypts containing abundant goblet cells and underlying lamina propria; section of human Dukes’ A colon cancer (**b**) with moderately-to-well-formed crypts lined by atypical epithelial cells which are large and tall; section of human Dukes’ B colon cancer (**c**) with the gland lumen containing cellular debris; section of human Dukes’ C and D (**d**–**f**) colon with irregularly and poorly formed glands lined by atypical epithelial cells with high mitotic activity and necrotic debris.

The normal adult colonic mucosa comprises surface columnar epithelium containing goblet cells, which also line the inner surface of the crypts within the lamina propria (Figure 1a). The nuclei of columnar and goblet cells are located at the basal ends of the cells. The muscularis mucosa borders with the submucosal connective tissue. In colorectal cancer (Dukes’ A and B), moderately-to-well-formed crypts or glands are observed, lined by atypical epithelial cells that are large and tall, featuring enlarged nuclei with prominent nucleoli (Figure 1b,c). These cells exhibit a loss of polarity and an increase in mitotic figures. Necrotic debris is visible inside the lumina of the crypts or glands, and there is a slight desmoplastic response of the stroma surrounding the tumor. In colorectal cancer (Dukes’ C and D), irregularly and poorly formed crypts or glands are present, lined by larger and taller atypical epithelial cells with larger nuclei and prominent nucleoli (Figure 1d–f). The loss of polarity is more pronounced, along with an increased number of mitotic figures. Necrotic debris is also evident within the lumen of adenocarcinomatous crypts or glands. Additionally, there is a desmoplastic response of the stroma surrounding the tumor.

### 3.2. Double Immunofluorescence Staining with PDE4B and SFRP5 Markers and Quantification of Immunoreactive Cells

**Figure 2:**
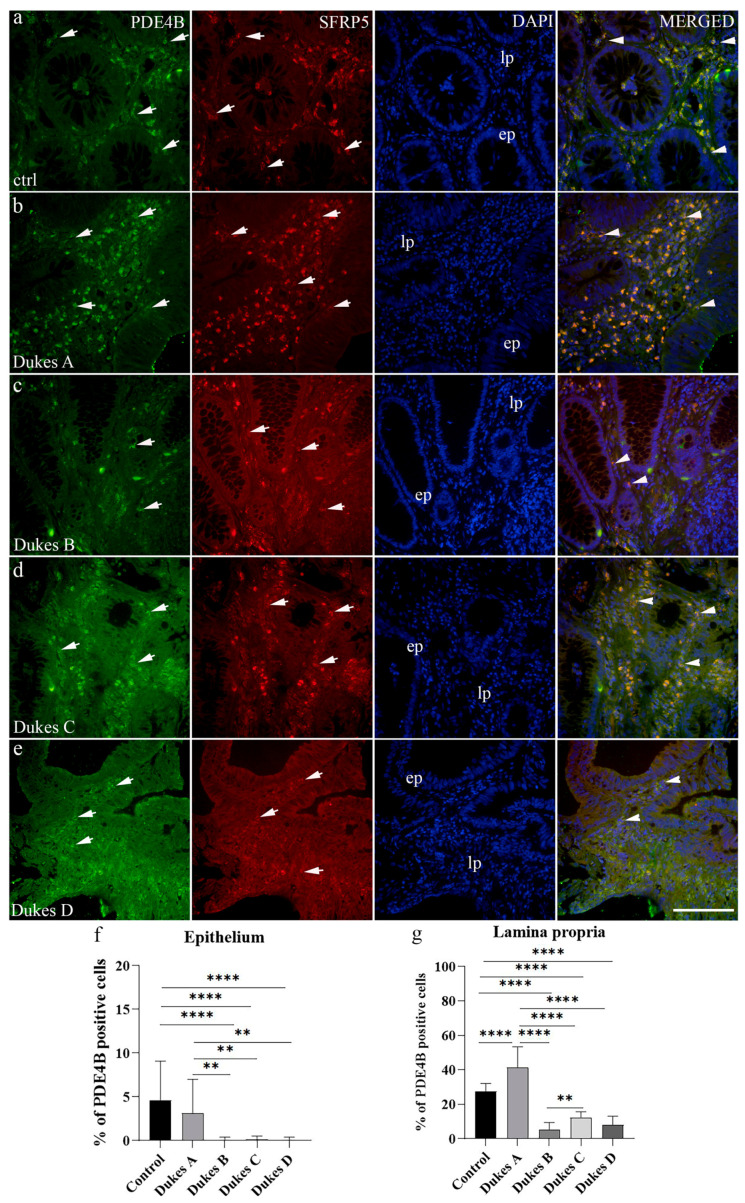
Immunofluorescence staining of the control normal adult human colon (**a**), human Dukes’ A (**b**), human Dukes’ B (**c**), human Dukes’ C (**d**), and human Dukes’ D (**e**) colon cancer. Arrows show the expression pattern of phosphodiesterase 4B (PDE4B) and secreted frizzled related protein 5 (SFRP5) positive cells in epithelium (ep) and lamina propria (lp) as indicated on the DAPI image. Co-localization of PDE4B and SFRP5 is indicated by arrowheads under column merged. Images were taken at a magnification of 40×. The scale bar is 100 µm, which refers to all images. The percentages of PDE4B-positive cells in control tissue and human Dukes’ A–D CRC stages in the epithelium (**f**) and lamina propria (**g**). To compare expression of analyzed protein in different substructures of colon mucosa between the observed groups, ordinary one-way ANOVA followed by Tukey’s multiple comparison tests was used. Data are presented as the mean ± SD (vertical line). Bars are presented in different color for easier distinction between groups. Significant differences are indicated by ** *p* ˂ 0.001 and **** *p* ˂ 0.00001. In each group, a minimum of ten representative images were assessed.

The healthy colorectal tissue control exhibited strong PDE4B reactivity in both the epithelium and lamina propria (Table 3). In the lamina propria, there were 27.47% PDE4B-positive cells, while in the epithelium, the percentage was 4.78% (Figure 2a,f). Co-localization of PDE4B and SFRP5 within the same cell was observed only in lamina propria (Figure 2a).

Similar to healthy colorectal tissue control, CRC stage Dukes’ A demonstrated strong expression of PDE4B in both epithelium and lamina propria (Table 3).

In the lamina propria, there were 42.64% of PDE4B-positive cells, while in the epithelium, the percentage was 2.82% (Figure 2b,f,g). Co-localization of PDE4B and SFRP5 in the same cell was observed in both lamina propria and epithelium (Figure 2b). CRC stage Dukes’ B exhibited strong expression of PDE4B in both epithelium and lamina propria (Table 3). In the lamina propria, there were 5.44% PDE4B-positive cells, while in the epithelium, the percentage was 0.06% (Figure 2c,f,g). Co-localization of PDE4B and SFRP5 in the same cell was observed only in lamina propria (Figure 2c). CRC stage Dukes’ C displayed strong expression of PDE4B in both epithelium and lamina propria (Table 3). In the lamina propria, there were 12.7% PDE4B-positive cells, while in the epithelium, the percentage was 0.13% (Figure 2d,f,g). Co-localization of PDE4B and SFRP5 in the same cell was observed in both lamina propria and epithelium (Figure 2d). CRC stage Dukes’ D had strong expression of PDE4B in both epithelium and lamina propria (Table 3). In the lamina propria, 8.56% of PDE4B-positive cells were present, while in the epithelium, the percentage was 0.06% (Figure 2e–g). Co-localization of PDE4B and SFRP5 in the same cell was observed only in lamina propria (Figure 2e).

**Figure 3:**
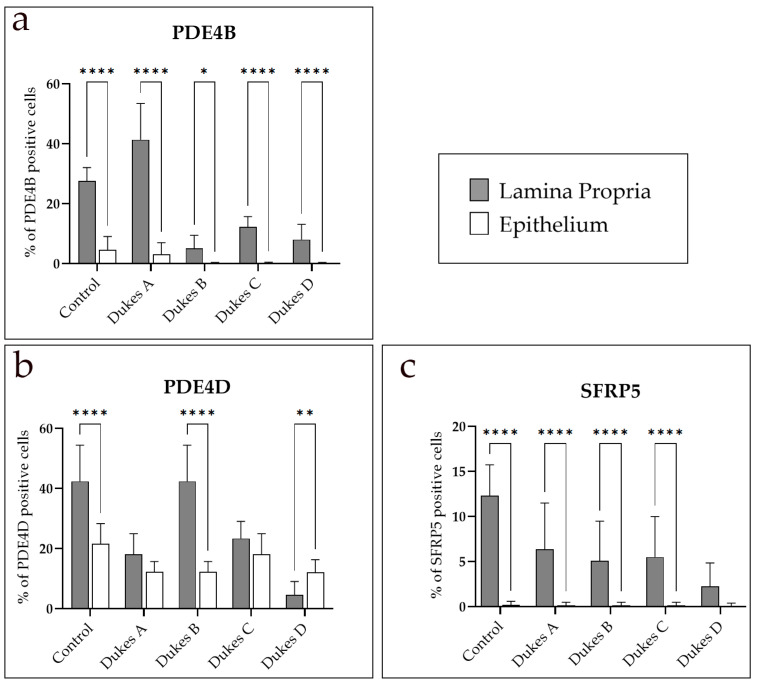
The percentages of phosphodiesterase 4B (PDE4B) (**a**), phosphodiesterase 4D (PDE4D) (**b**), and secreted frizzled related protein 5 (SFRP5) (**c**) positive cells in the epithelium and lamina propria in control tissue and human Dukes’ A–D colon cancer. Within each analyzed group, a two-way ANOVA test followed by Šídák’s multiple comparisons test was used to compare the expression of the observed proteins between the different substructures. Data are presented as the mean ± SD (vertical line). Significant differences are indicated by * *p* ˂ 0.01, ** *p* ˂ 0.001, and **** *p* ˂ 0.00001. In each group, a minimum of ten representative images were assessed.

The expression of PDE4B between the lamina propria and epithelium in the observed tissues revealed a statistically significant increase in the PDE4B-positive cells in the lamina propria compared to the epithelium in all examined tissues (*p* < 0.05) (Figure 3a).

Statistically significant differences were observed in the number of PDE4B-positive epithelial cells between control and CRC stage Dukes’ B, C, and D, as well as between CRC stage Dukes’ A and CRC stages Dukes’ B, C, and D (Figure 2f). Regarding the number of PDE4B-positive cells in lamina propria, a statistically significant difference was observed between control and all CRC Dukes’ stages, as well as CRC stage Dukes’ A and CRC stage Dukes’ B, C, and D. Additionally, CRC stage Dukes’ C had significantly more PDE4B-positive cells in the lamina propria than in the CRC stage Dukes’ B (Figure 2f).

### 3.3. Double Immunofluorescence Staining with PDE4D and SFRP5 Markers and Quantification of Immunoreactive Cells

**Figure 4:**
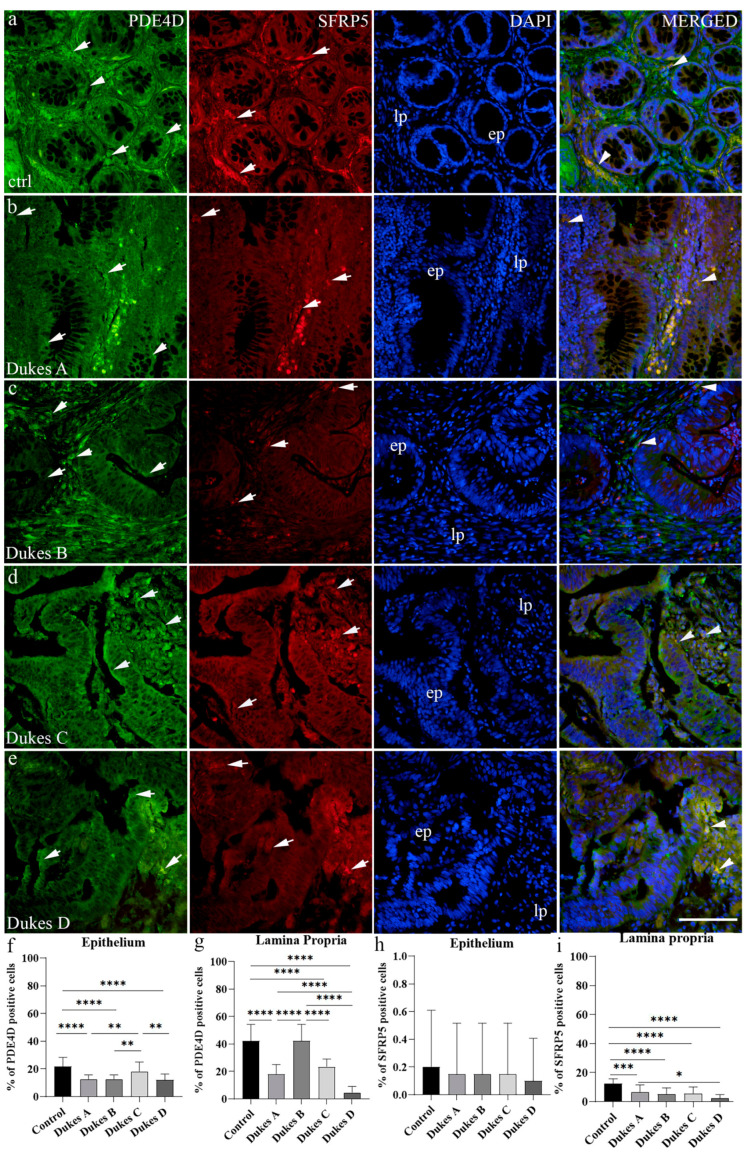
Immunofluorescence staining of the control normal adult human colon (**a**), human Dukes’ A (**b**), human Dukes’ B (**c**), human Dukes’ C (**d**), and human Dukes’ D (**e**) colon cancer. Arrows show the expression pattern of phosphodiesterase 4D (PDE4D) and secreted frizzled related protein 5 (SFRP5) positive cells in epithelium (ep) and lamina propria (lp) as indicated on the DAPI image. Co-localization of PDE4D and SFRP5 is indicated by arrowheads under column merged. Images were taken at a magnification of 40×. The scale bar is 100 µm, which refers to all images. The percentages of PDE4D- and SFRP5-positive cells in control normal adult human colon and human Dukes’ A–D colon cancer in the epithelium (**f**,**h**) and lamina propria (**g**,**i**). To compare expression of analyzed proteins in different substructures of colon mucosa between the observed groups, ordinary one-way ANOVA followed by Tukey’s multiple comparison tests was used. Data are presented as the mean ± SD (vertical line). Bars are presented in different color for easier distinction between groups. Significant differences are indicated by * *p* ˂ 0.01, ** *p* ˂ 0.001, and **** *p* ˂ 0.00001. In each group, a minimum of ten representative images were assessed.

The healthy colorectal tissue control exhibited strong reactivity of PDE4D and SFRP5 in both the epithelium and lamina propria (Table 3), with a similar percentage of positive cells. Namely, in the lamina propria, there were 27.5% and 26.8% of PDE4D- and SFRP5-positive cells, while in the epithelium, the percentages were 4.57% and 4.82%, respectively (Figure 4a,f,g). Co-localization of PDE4D and SFRP5 in the same cell was observed only in the lamina propria (Figure 4a).

Similar to healthy colorectal tissue control, CRC stage Dukes’ A demonstrated moderate expression of PDE4D and SFRP5 in the epithelium and strong PDE4D and SFRP5 in the lamina propria (Table 3). In the lamina propria, there were 42.22% and 40.93% of PDE4D- and SFRP5-positive cells, while in the epithelium, the percentages were 2.27% and 2.33%, respectively (Figure 4b,f,g). Co-localization of PDE4D and SFRP5 in the same cell was observed in both the lamina propria and epithelium (Figure 4b). CRC stage Dukes’ B exhibited mild expression of PDE4D in the epithelium and strong expression in the lamina propria, while SFRP5 displayed moderate expression in the lamina propria and strong expression in the epithelium (Table 3). In the lamina propria, there were 5.27% and 5.56% of PDE4D- and SFRP5-positive cells, respectively, while in the epithelium, the percentages were the same at 0.07% (Figure 4c,f,g). Co-localization of PDE4D and SFRP5 in the same cell was observed only in the lamina propria (Figure 4c). CRC stage Dukes’ C displayed moderate expression of PDE4D in both the epithelium and lamina propria, while SFRP5 displayed strong expression in both structures (Table 3). In the lamina propria, there were 12.48% and 12.73% of PDE4D- and SFRP5-positive cells, while in the epithelium, the percentages were 0.07% and 0.08%, respectively (Figure 4d,f,g). Co-localization of PDE4D and SFRP5 in the same cell was observed in both the lamina propria and epithelium (Figure 4d).

CRC stage Dukes’ D had mild expression of PDE4D in both the epithelium and lamina propria, while SFRP5 displayed mild expression in the epithelium and moderate expression in the lamina propria (Table 3). In the lamina propria, there were 8.6% and 8.5% of PDE4D- and SFRP5-positive cells, respectively, while in the epithelium, the percentages were the same at 0.07% (Figure 4e–g). Co-localization of PDE4D and SFRP5 in the same cell was observed only in the lamina propria (Figure 4e).

The expression of PDE4D between the lamina propria and epithelium demonstrated a statistically significant increase in the percentage of PDE4D-positive cells in control tissues as well as in Dukes’ B and Dukes’ D stages of CRC (*p* < 0.0001 and *p* < 0.01, respectively) (Figure 3b). No significant differences were detected in the Dukes’ A and Dukes’ C stages (Figure 3b).

Statistically significant differences were observed in the number of PDE4D-positive epithelial cells between control and CRC stage Dukes’ A, B, and D, as well as between CRC stage Dukes’ C and CRC stage Dukes’ A, B, and D (Figure 4f). Concerning the number of PDE4D-positive cells in lamina propria, a statistically significant difference was observed between control and CRC stage Dukes’ A, C, and D, as well as CRC stage Dukes’ B and CRC stage Dukes’ A, C, and D. Additionally, CRC stage Dukes’ A had significantly more PDE4D-positive cells in the lamina propria than in the CRC stage Dukes’ D (Figure 4f).

Regarding the expression of SFRP5 in the lamina propria compared to the epithelium, a statistically significant increase in the percentage of SFRP5-positive cells was noticed in the lamina propria in all examined tissues (*p* < 0.0001) except for Dukes’ D, where no significant difference was found (Figure 3c). Statistically significant differences were observed in the number of SFRP5-positive cells in the lamina propria between control and all CRC stages, as well as between CRC stage Dukes’ A and CRC stage Dukes’ D (Figure 4i). In the epithelium, there was no statistically significant difference between groups (Figure 4h).

### 3.4. Differential Expression

**Figure 5:**
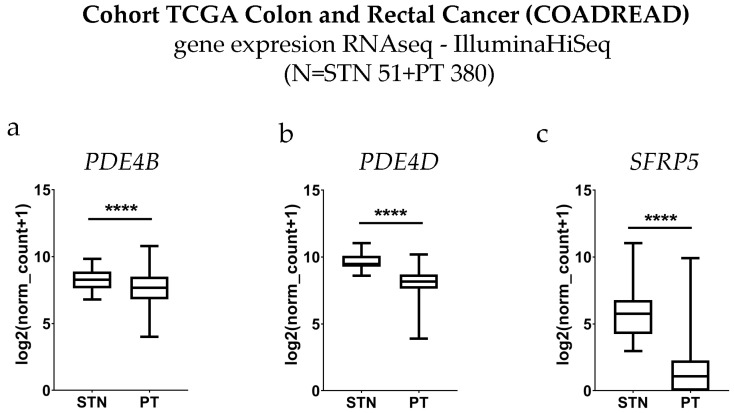
Cohort TCGA colon and rectal cancer (COADREAD) gene expression of phosphodiesterase 4B (*PDE4B*) (**a**), phosphodiesterase 4D (*PDE4D*) (**b**), and secreted frizzled related protein 5 (*SFRP5*) (**c**) RNAseq—IlluminaHiSeq with control (STN) and colorectal cancer patient groups (PT). Data are expressed as log2(norm count + 1), unpaired *t*-test with Welch’s correction, **** *p* < 0.0001.

We extracted CRC tumor tissue and control tissue RNA-sequencing data from the COADREAD study. Gene expression of all three studied genes, PDE4B (*p* < 0.0001), PDE4D (*p* < 0.0001), and SFRP5 (*p* < 0.0001), was significantly lower in tumor tissue in comparison to the control tissue (Figure 5).

### 3.5. Survival Analysis

**Figure 6:**
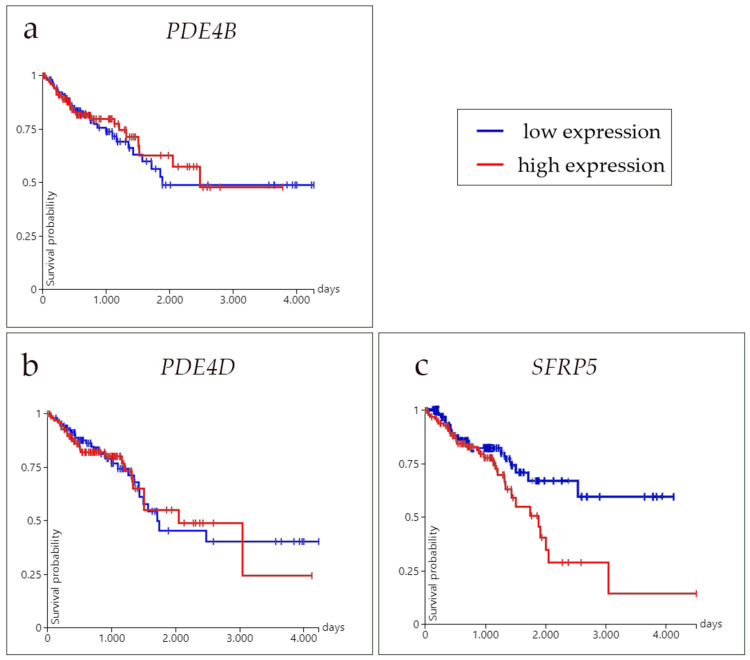
Graphic presentation of survival analysis (days) of phosphodiesterase 4B (*PDE4B*) (**a**), phosphodiesterase 4D (*PDE4D*) (**b**), and secreted frizzled related protein 5 (*SFRP5*) (**c**) in high (red line) and low (blue line). mRNA expression in colorectal cancer was expressed as the average survival time in days. The Kaplan–Meier method and log-rank test for survival length were used. Data are used from the TCGA Colon and Rectal Cancer (COADREAD) study.

The survival rates concerning the high and low mRNA expressions of PDE4B, PDE4D, and SFRP5 in CRC were analyzed (Figure 6).

There was no significant difference in survival times in colorectal carcinoma between the high- and low-expression groups of PDE4B and PDE4D (*p* = 0.6966 and *p* = 0.8914, respectively). However, a statistically significant difference (*p* = 0.03824) in survival times was found between the high and low SFRP5 expression groups, where low expression of the SFRP5 denoted better survival probability (Figure 6).

## 4. Discussion

As the phosphodiesterase enzyme family (PDEs) plays a crucial role in signal transduction, it displays promising pharmacological targets in a variety of diseases. Due to the lack of biomarkers for early diagnosis, tumor resection is frequently delayed until the disease has progressed significantly, leaving radiotherapy and chemotherapy as the primary treatment options. PDE4B and PDE4D have recently been reported as oncogenes in various human cancers. They play critical roles in cancer progression by degrading cAMP. This degradation influences cancer development by promoting cell proliferation, reducing apoptosis, and facilitating metastasis through pathways such as phosphatidylinositol-3 kinase/protein kinase B/mammalian target of rapamycin (PI3K/AKT/mTOR) and mitogen-activated protein kinases (Ras/Raf-MEK-MAPKs) [25,26,27,28]. PDE4B has been shown to contribute to a pro-inflammatory environment within tumors, which can support tumor growth and immune evasion [26,28]. PDE4D, on the other hand, can alter gene expression and impact the tumor microenvironment, thereby influencing various aspects of cancer biology, including the response to therapies [26]. Additionally, SFRP5 acts as a soluble modulator of Wnt signaling by binding to Wnt proteins and preventing them from interacting with their receptors. It also acts as a tumor suppressor, preventing uncontrolled cell growth and differentiation. It also has anti-inflammatory properties and can inhibit epithelial-mesenchymal transition (EMT), reducing metastasis [29,30].

Together, these genes impact tumor initiation, progression, and metastasis through distinct but interrelated mechanisms. However, their expression and significance in CRC have not been elucidated. Therefore, we investigated the protein and gene expression patterns of *PDE4B*, *PDE4D*, and *SFRP5* to provide valuable insights into their involvement in CRC.

Increased PDE4B expression correlates with relapsed colorectal cancer cell lines, suggesting its potential as a prognostic molecular marker in CRC [31]. Pleiman et al. found that loss of PDE4B function in the ApcMin/+ mouse significantly increases the number of colonic adenomas [32]. In this context, they proposed that a feedback mechanism could explain the protective role of PDE4B. Similarly, our result showed the highest PDE4B expression in the control tissue and Dukes’ A stage. According to this model, PDE4B is initially triggered by early oncogenic stress related to cAMP. However, as observed in cases of advanced human colon cancer, it is subsequently deactivated through epigenetic suppression. Mahmood et al. discovered a higher expression of PDE4B in patients with colorectal neoplasia [33] and proposed that overexpression of PDE4B appears as a malfunctioning protein in the noncancerous mucosa of the colon. This finding suggests a tendency toward reduced *PDE4B* activity in the colon mucosa of patients with colorectal neoplasia. These results follow our findings of lower *PDE4B* expression in the mucosa of CRC Dukes’ stages B, C, and D as well as Illumina Hi-Seq COADREAD TCGA survival analysis data.

Strong evidence suggests that PDE4D contributes to tumor advancement in lung, central nervous system (CNS), and skin cancers. Additionally, certain isoforms of PDE4D exhibit contrasting functions in prostate cancer [28].

Kim et al. suggested that PDE4D has been implicated in the development and progression of CRC [34]. Nummela et al. found that overexpression of PDE4D leads to inhibition of the proliferation of CRC cells through PDE4 inhibitors [35]. In our study, PDE4D expression was lowest in the Dukes’ D stage compared to the controls and other Dukes’ stages. However, this might be tissue-specific since the finding was the opposite in pancreatic ductal adenocarcinoma (PDAC) [36]. Namely, Liu et al. found that high expression of PDE4D correlates with poor prognosis and clinical progression of PDAC. PDE4D has also been implicated in other cancers, such as prostate cancer, medulloblastoma, and leukemia, indicating that PDE4D may be a more general target for cancer therapy [37].

Our finding suggests that a lower level of PDE4D, as in the CRC stage Dukes’ D, will lead to a worse CRC prognosis in regards to metastatic spreading of the disease. Illumina Hi-Seq COADREAD TCGA data regarding survival analysis also confirm the poorer prognosis in patients with a lower *PDE4D* expression. Blocking PDE4D affects the levels of protein kinase A (PKA), sirtuin 1 (Sirt1), protein kinase B (Akt), and BCL2 apoptosis regulator/apoptosis regulator BAX (Bcl-2/Bax), which are involved in signaling pathways that regulate endocrine reactions, stress resilience, neuronal autophagy, and cell death [36]. These studies imply that PDE4D is aberrantly expressed in CRC cells and patients with CRC, which may contribute to the malignant phenotype. However, further studies are required to fully understand the mechanisms underlying PDE4D overexpression in CRC and develop targeted therapies.

In our study, SFRP5 expression was lowest in stage Dukes’ D. Our immunohistochemistry and RNA sequencing data results align with Kirana et al., where analysis of an independent tissue cohort from The Cancer Genome Atlas database on 637 patients revealed significantly lower *SFRP5* RNA expression in CRC tumor tissue compared with adjacent normal mucosa. The same study found that the levels of SFRP5 were significantly lower in CRC patients with either vascular invasion or liver metastasis, which corresponds to stage Dukes’ D [38]. At the same time, they also found that a high serum level of circulating SFRP5 (cSFRP5) is associated with longer disease-free survival [38]. The study of Huang et al. noted that the mRNA levels of *SFRP5* were significantly downregulated in 80% of CRC [39]. Additionally, Liu et al. also found that expression levels of *SFRP5* were reduced in gastric cancer [14]. This finding implies that a higher level of *SFRP5* might predict longer disease-free survival and, therefore, might serve as a prediction marker for CRC. This finding opposes the Illumina Hi-Seq COADREAD TCGA survival analysis data on *SFRP5* expression analyzed in our study, where we noted better prognosis in patients with lower *SFRP5* expression.

Additionally, SFRP5 hypermethylation has been associated with the risk of colorectal cancer, and this hypermethylation can lead to the activation of the Wnt signaling pathway, which is a key driver of CRC progression [40]. Studies also show that, in colorectal cancer, kidney cancer, and breast and prostate cancer, a decrease in the level of SFRP, but also an increase in the level of SFRP expression, depending on the degree of malignancy, has been observed. This indicates the probability that high concentrations inhibit and low concentrations potentiate Wnt signaling [41].

We also analyzed the expression levels of *PDE4B*, *PDE4D*, and *SFRP5* genes in CRC tissues compared to control tissues using RNA-sequencing data from the UCSC Xena browser. The findings reveal significant differences in the expression of these genes between CRC patients and healthy controls, with notable implications for patient prognosis. Namely, our results demonstrate that *PDE4B*, *PDE4D*, and *SFRP5* are significantly under-expressed in CRC tissues compared to control tissues. The Kaplan–Meier survival analysis and the log-rank (Mantel–Cox) test revealed distinct prognostic implications based on the expression levels of *PDE4B*, *PDE4D*, and *SFRP5*. Patients with low levels of *PDE4B* and *SFRP5* exhibited significantly longer overall survival compared to those with low levels of *PDE4D*. This finding underscores the differential impact of these genes on CRC patient outcomes. The data align with our immunohistochemical results and might suggest a potential tumor-suppressive role for these genes in CRC. The reduced expression of these genes in tumor tissues may indicate their involvement in disrupted pathways during colorectal carcinogenesis.

At present, there are no FDA-approved cancer treatments that target PDE4B, PDE4D, or SFRP5. Nevertheless, as previously mentioned, research suggests that PDE4B and PDE4D are significant contributors to the progression of cancer and inflammation [42,43]. The relationship between chronic inflammation and cancer development has been widely recognized. Chronic inflammation may establish a tumor-promoting environment by continuously producing pro-inflammatory cytokines and other mediators. This process can result in DNA damage and genomic instability, which are critical factors in the initiation and progression of cancer [44,45]. For example, chronic inflammation has been linked to the development of numerous malignancies, such as chronic bronchitis and lung cancer [46], as well as the induction of bladder cancer [47]. The risk of lung and colorectal malignancies is considerably elevated by conditions such as chronic bronchitis and inflammatory bowel diseases, including ulcerative colitis and Crohn’s disease [46,48]. The critical role of inflammation in the etiology of cancer is further evidenced by the observation that the incidence of cancer can be reduced by managing inflammatory conditions with anti-inflammatory agents [44,45,49]. Consequently, the comprehension of the cellular mechanisms that underlie inflammation-induced cancer is an essential area of ongoing research [45]. PDE4 inhibitors, including roflumilast and apremilast, used at the present to treat inflammatory conditions, are currently being investigated for their potential in cancer therapy. These inhibitors have the potential to inhibit tumor growth and enhance the efficacy of other treatments [42,43]. Furthermore, preclinical studies have demonstrated that PDE4D inhibitors can potentially restrict prostate cancer cell proliferation [50]. Ongoing research is being conducted on SFRP5, a modulator of the Wnt signaling pathway. This pathway is essential for cancer progression, and therapies designed to restore or replicate SFRP5 activity are currently being evaluated for their potential to treat cancer [51]. These targets hold promise for future cancer treatments as research continues to advance.

Our study lacks detailed information on macroscopic examination and measurements, such as tumor size, and morphological characteristics. This limitation arises from the variability within our patient cohort: some patients did not undergo surgical operations, some had surgeries at different facilities, and others sought treatment abroad for better healthcare standards. These factors significantly limit our ability to provide comprehensive data.

## 5. Conclusions

In conclusion, our results emphasize the potential clinical utility of *PDE4B*, *PDE4D*, and *SFRP5*, genes that impact tumor initiation, progression, and metastasis, as biomarkers for CRC. Considering significantly lower gene expression, aligned with our immunohistochemical data in tumor tissue in comparison to the control tissue, as well as the significantly poorer survival rate in the case of its higher expression, we can hypothesize that *SFRP5* is the most promising biomarker for CRC out of the observed proteins. Assessing their expression levels could provide valuable prognostic information and help open new avenues for biomarker-driven diagnosis and therapy. For instance, targeting pathways affected by these genes might offer new avenues for CRC treatment. Furthermore, the significant correlation between gene expression levels and patient survival suggests that therapeutic modulation of *PDE4B*, *PDE4D*, and *SFRP5* activity could improve patient outcomes. This highlights the need for further studies to explore targeted therapies that can modulate these pathways effectively. However, further research is needed to elucidate the potential correlations between PDE4B, PDE4D, and SFRP5 and the signaling network in CRC.

## Figures and Tables

**Table 1:** Patients’ characteristics according to CRC stages.

Histology and Stage		No Patients (n)	Sex (n)	Age (Year)
CRC	Dukes’ A	12	M (6)	56.83 ± 6.15
			F (6)	51.83 ± 5.87
	Dukes’ B	11	M (5)	75.8 ± 4.07
			F (6)	82.17 ± 7.77
	Dukes’ C	10	M (8)	69 ± 9.46
			F (2)	69 ± 7.07
	Dukes’ D	10	M (7)	66.29 ± 10.20
			F (3)	61.33 ± 7.72

CRC—colorectal cancer; M—male; F—female. Values are means ± standard deviations.

**Table 2:** Primary and secondary antibodies used for immunohistochemistry.

Primary Antibody/Dilution	Secondary Antibody/Dilution
PDE4B goat polyclonal antibody, Invitrogen (PA5-18473)/1:100	Alexa Fluor 488-conjugate AffiniPure DonkeyAnti-Sheep IgG (H+L), Jackson Laboratories 713-545-003/1:300
PDE4D goat polyclonal antibody, Invitrogen (PA5-18459)/1:100	Alexa Fluor 488-conjugate AffiniPure DonkeyAnti-Sheep IgG (H+L), Jackson Laboratories 713-545-003/1:300
SFRP5 rabbit polyclonal antibody, Invitrogen (PA5-30169)/1:200	Rhodamine (TRITC)-conjugated AffiniPure DonkeyAnti-Rabbit IgG (H+L), Jackson Laboratories 711-025-152/1:300

**Table 3:** Immunoreactivity to PDE4B, PDE4D, and SFRP5 markers in colorectal cancer (CRC) through Dukes’ stages A-D and control tissue.

Structure	Antibodies														
	PDE4B					PDE4D					SFRP5				
	Ctrl	A	B	C	D	ctrl	A	B	C	D	ctrl	A	B	**C**	**D**
epithelium	+++	+++	+++	+++	+++	+++	++	+	++	+	+++	++	+++	+++	+
lamina propria	+++	+++	+++	+++	+++	+++	+++	+++	++	+	+++	+++	++	+++	++

+++ strong reactivity; ++ moderate reactivity; + mild reactivity. ctrl—control, A—Dukes’ A, B—Dukes’ B, C—Dukes’ C, D—Dukes’ D.

## Data Availability

The raw data supporting the conclusions of this article will be made available by the authors on request.
